# Enhanced bone cement distribution in percutaneous vertebroplasty using a curved guide wire: a propensity score matching analysis

**DOI:** 10.1186/s12891-024-07951-8

**Published:** 2024-10-22

**Authors:** Xuyan Hu, Zijin Zhang, Yisong Yang, Gang Zhang, Shen Cao, Bing Yu, Yubing Zhang

**Affiliations:** 1 Anhui No. 2 Provincial People’s Hospital Clinical College of Anhui Medical University, No. 1868 Dangshan Road, Yaohai District, Hefei, Anhui 230000 China; 2 The Fifth Clinical Medical College of Anhui Medical University, Hefei, Anhui 230000 China; 3 Department of Spine Surgery, Anhui No. 2 Provincial People’s Hospital, No. 1868 Dangshan Road, Yaohai District, Hefei, Anhui 230000 China; 4 School of Engineering, Newcastle University, Newcastle upon Tyne, NE1 7RU UK; 5 Graduate Department, Bengbu Medical University, Bengbu, Anhui 233000 China

**Keywords:** Curved guide wire, Percutaneous vertebroplasty, Osteoporotic vertebral compression fractures, Pain relief, Functional recovery, Bone cement distribution

## Abstract

**Background:**

Osteoporotic vertebral compression fractures (OVCF) severely affect the quality of life in the aged population. Percutaneous vertebroplasty (PVP) alleviates pain and stabilizes vertebrae, but suboptimal bone cement distribution can cause complications. Hence, this study aimed to clarify whether a new technique for PVP, using a curved guide wire, enhances the distribution of bone cement and improves clinical outcomes in patients with OVCF.

**Methods:**

Patients with single-segment OVCF underwent PVP or curved guide wire percutaneous vertebroplasty (C-PVP). Propensity score matching (PSM) was employed to balanced the baseline characteristics. The primary outcomes were the visual analog scale (VAS) and Oswestry disability index (ODI) scores. The secondary outcomes included assessments of bone cement distribution, bone cement injection volume, radiological parameters, and general clinical results. Additionally, Complications and adverse events were documented.

**Results:**

After PSM analysis, each group comprised 54 patients, which significantly reduced baseline differences. The C-PVP group showed better clinical outcomes compared to the traditional PVP group. One month after surgery, the C-PVP group had significantly lower VAS and ODI scores (*p* < 0.001). These improvements persisted at six months and the final follow-up. Additionally, bone cement distribution scores were better (*p* < 0.001), injection volume was higher (*p* = 0.03), leakage was less frequent (*p* = 0.02), and adjacent vertebral fractures occurred less frequently (*p* = 0.04) in the C-PVP group. Radiological parameters and overall clinical outcomes revealed no significant differences between the two groups.

**Conclusion:**

The use of curved guide wire in PVP significantly improves bone cement distribution and injection volume, resulting in better clinical efficacy in patients with OVCF.

## Introduction

Osteoporotic Vertebral Compression Fractures (OVCF) is a severe public health issue, especially for the aged population [1]. Research shows that the occurrence of OVCF is significantly higher in women compared to men, at about 1.2% per year in women and 0.4% per year in men [2]. OVCF often leads to chronic pain, impaired mobility, and an increased risk of subsequent fractures [3]. These fractures directly impact daily activities and overall well-being, leading to long-term morbidity and dependence in self-care activities. The back pain caused by these fractures can be so debilitating that even simple activities of daily living become difficult for patients to perform, decreasing their quality of life [4]. Given the severity of OVCF’s consequences, improving treatment efficacy is crucial.

Percutaneous vertebroplasty (PVP) is universally regarded as the treatment modality of choice for OVCF [5]. This procedure is performed through the skin by injecting bone cement into the compressed vertebra, thereby reinstating its height and strength. It is less invasive, with a smaller incision, making it ideal for aged and comorbid patients who cannot tolerate open surgery. Research indicates that PVP is highly beneficial in alleviating pain and disability associated with OVCF, playing a significant role in the management of this condition [6]. Furthermore, PVP increases the overall quality of life of the patient by reducing disability and improving functional recovery [7]. Additional clinical trials have demonstrated that PVP significantly decreases mortality rates in patients with OVCF compared to non-surgical management, providing both immediate and long-term survival benefits [8]. However, suboptimal bone cement distribution in PVP may lead to insufficient stabilization of the vertebral body, which may cause inadequate pain relief, an increased risk of refracture, and potentially contribute to progressive vertebral collapse and kyphotic deformity [9, 10]. In such cases, non-uniform distribution means the overall success of the procedure is minimal for the patients [11]. In contrast, improved bone cement distribution lowers the incidence of complications, including leakage of cement and subsequent fractures, and provides better structural stability and pain relief [12]. Still, surgical techniques that achieve better bone cement distribution affordably and feasibly are lacking today. Herein, we introduce a new surgical procedure that improves bone cement distribution using a curved guide wire. We conducted a retrospective study to compare this new surgical technique with traditional PVP.

## Materials and methods

### Study design

This study was a retrospective analysis utilizing a cohort design at a single center. Propensity score matching (PSM) was employed to balance the differences in baseline characteristics. The study was conducted at Anhui No. 2 Provincial People’s Hospital between September 2021 and December 2023. Patients with a clinical diagnosis of single-segment OVCF (T5-L5) were included. Relevant laboratory tests and radiographic examinations were performed. The study was approved by the Clinical Ethics Committee of Anhui No. 2 Provincial People’s Hospital in accordance with the Helsinki Principles (Date: 07.06.2024; Approval No. R2024-071).

### Inclusion and exclusion criteria

The criteria for inclusion were as follows: (1) Patients’ ages ranged from 55 to 80 years; (2) Single-segment, acute thoracolumbar OVCF (T5-L5), confirmed by preoperative spinal imaging via X-ray, CT, and MRI, with kyphosis Cobb angle ranging between 10° and 40°; (3) OVCF had to be of recent origin, within the past four weeks; (4) Back pain VAS score ≥ 5; (5) BMD T-value ≤ -2.5; (6) All patients must have consented to the treatment. The criteria for exclusion were as follows: (1) Presence of bone tumors or myeloma; (2) History of spinal surgery at the same or adjacent vertebral levels; (3) Patients with multiple vertebral fractures or recurrence at the same vertebral level; (4) Unwillingness or inability to comply with postoperative follow-up requirements; (5) Osteomyelitis, discitis, or active systemic infection; (6) Spinal cord or nerve root compression syndrome; (7) Severe systemic comorbidities.

### Study population

**Fig. 1:**
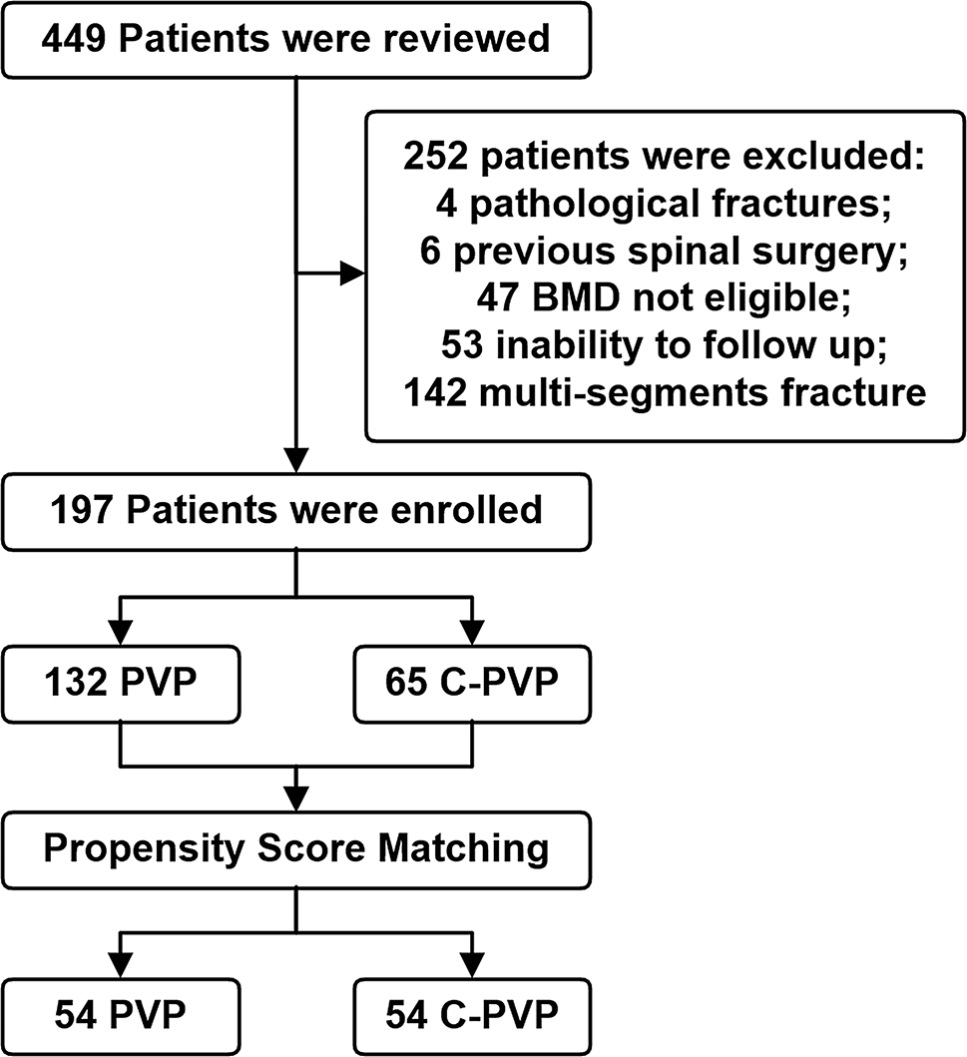
Flowchart of the study

In our study, we enrolled a total of 197 patients (Fig. 1). Among these, 132 patients underwent PVP, and 65 patients underwent curved guide wire percutaneous vertebroplasty (C-PVP). The baseline characteristics (sex and age) of the original PVP and C-PVP groups were significantly different. Consequently, the PSM method was applied to equalize the differences between the two groups. Patients in the C-PVP group were matched with counterparts in the PVP group based on baseline characteristics. After PSM, no significant differences were observed in the baseline characteristics between the two groups, and 54 patients were included in each group for further analysis.

### Surgical procedure

Under fluoroscopic guidance, experienced surgeons performed all procedures with patients positioned prone on a radiolucent table. Bone-biopsy needles were inserted percutaneously via a transpedicular approach into the fractured vertebral body, entering 1–2 cm lateral to the outer wall of the pedicle, and were then advanced obliquely toward the anterior third of the vertebral body. The inner needle cores of bone-biopsy needles were removed, followed by the insertion of guide wires and placement of working cannulas over them.

In the PVP group, the guide wires were removed, and high density polymethylmethacrylate (PMMA) bone cement was injected through the cannulas under fluoroscopic control. Injection ceased when the cement approached the posterior vertebral wall or if leakage was detected.

**Fig. 2:**
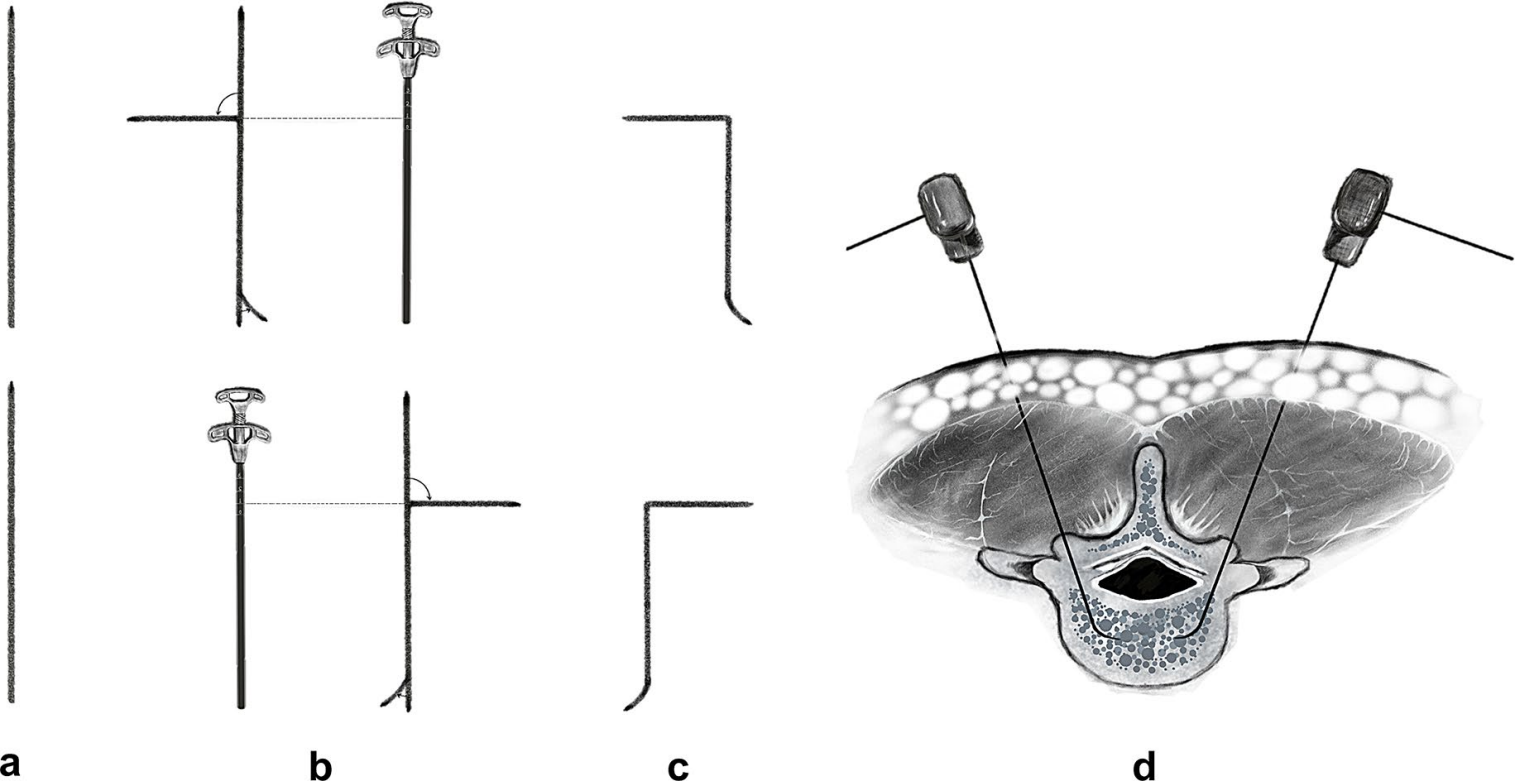
Schematic diagram: (**a**) Straight guide wires. (**b**) Guide wires during the bending process: In the top part, using the mark on the bone cement injector as a reference point, the guide wire is bent to 90°; in the bottom part, the guide wire is bent into a circular arc with an approximate radius of 1 cm. (**c**) Curved guide wires. (**d**) Guide wires are inserted into the vertebral body

In the C-PVP group, the guide wires were removed and modified into curved shapes as depicted in Fig. 2. To accommodate variations in surgical instrument brands across regions, the proximal end of each guide wire was bent to a 90° angle at a reference point determined by inserting the bone cement injector into the working cannula and recording the lowest point protruding from the cannula. The distal end was bent into a circular arc with an approximate radius of 1 cm. Because the guide wires were made of Nitinol, a material with excellent shape memory properties, they automatically resumed their curved shapes upon reinsertion through the working cannulas into the vertebral bodies. This configuration allowed for controlled manipulation within the vertebral bodies, enabling the creation of multiple dispersion channels around the fracture sites without breaching the cortical bone of the vertebra. After the curved guide wires were removed, bone cement was injected through the working cannulas under continuous fluoroscopic monitoring, filling the dispersion channels created by the curved guide wires. Injection was halted when the cement neared the posterior vertebral wall or if leakage was detected.

After the cement solidified, instruments were withdrawn, and incisions were closed with sterile dressings. Postoperatively, all patients received osteoporosis medications. Vital signs and neurological status were closely monitored, and early mobilization was encouraged from the first postoperative day. Follow-up radiographs within one day assessed bone cement distribution and verified vertebral stability.

### Outcome and outcome assessments

#### Baseline characteristics assessment

Baseline characteristics were collected for demographic analysis, including the total patient count, age, sex, body mass index (BMI), bone mineral density (BMD) T-value, fracture type (Genant classification) [13], fracture segment, and comorbidities such as coronary artery disease (CAD), hypertension (HTN), chronic obstructive pulmonary disease (COPD), diabetes mellitus (DM), and neurologic disorders.

#### Primary outcome

The primary outcomes of this study were the visual analog scale (VAS) and Oswestry Disability Index (ODI) scores, which were assessed pre-surgery and at multiple post-surgery time points. The VAS score assessed pain relief, and the ODI score evaluated functional recovery. The final follow-up time varied from 6 to 32 months, which could have introduced time-confounding effects. This was addressed using Kaplan-Meier event-time curves to analyze the trend over time properly. The VAS scores can be classified into two groups: VAS ≤ 1 for complete pain relief and VAS > 1 for incomplete pain relief. Similarly, the ODI scores can be categorized into two groups: ODI ≤ 10 for complete functional recovery and ODI > 10 for incomplete functional recovery.

#### Secondary outcome

Secondary outcomes were evaluated through a comprehensive set of radiological parameters, bone cement distribution, and general clinical outcomes. The radiological parameters, including local kyphosis angle (LKA), anterior vertebral height ratio (AVHR), middle vertebral height ratio (MVHR), and posterior vertebral height ratio (PVHR), were measured pre-surgery and 24 h post-surgery to monitor vertebral changes and alignment. The vertebral height ratio is calculated by dividing the fractured vertebra height by the average height of the adjacent intact vertebrae. Bone cement distribution was also assessed and documented according to the radiographic aspect from 0 to 8 [14]. A crossline is drawn at the center of the vertebral body on both the anteroposterior and lateral radiographs, creating four quadrants in each view of the fractured vertebra. Cement spread involving more than one-third of the quadrant is considered satisfactory and scores 1 point. The maximum score can thus be between 0 and 8 points. General clinical data included time to final follow-up, operative time, the number of C-arm fluoroscopy frequencies, bone cement injection volume, adjacent vertebral fractures, and incidents of cement extravasation, categorized as types B, C, and S [15]. Adjacent vertebral fractures were evaluated during the final follow-up telephone call by asking about the occurrence of new or worsening symptoms, as well as any radiological findings that confirmed or indicated such fractures.

### Statistical analysis

Since baseline characteristics differed between eligible participants in the two groups, a PSM procedure was utilized to select a cohort of patients with comparable baseline characteristics. A 1:1 matching protocol was utilized, employing the nearest-neighbor algorithm for pairing. The caliper width was defined as 0.2 times the standard deviation of the logit of the propensity score.

Normality of continuous variables was assessed using the Kolmogorov-Smirnov test. Before PSM, baseline characteristics were evaluated using independent sample t-tests for continuous variables that followed a normal distribution, the Mann-Whitney U test for non-normally distributed continuous variables, and the Chi-square or Fisher’s exact tests for categorical data. Ordinal data were evaluated using the Mann-Whitney U test. After PSM, paired sample t-tests were applied to normally distributed continuous variables, the Wilcoxon test for non-normally distributed continuous variables and ordinal data, and McNemar’s tests for nominal data. Kaplan-Meier survival analysis was employed to evaluate the time to event for pain relief and functional recovery, with the log-rank test used for group comparisons. Statistical significance was defined as *p* < 0.05. All analyses were conducted using SPSS version 26.0 (IBM Corp., Armonk, NY, USA) and R version 4.2.3 (R Foundation for Statistical Computing, Vienna, Austria).

## Results

### Baseline characteristics assessment

Table 1 shows that, before PSM, significant differences existed between the C-PVP and PVP groups in the distribution of sex and the BMD T-values. After matching, the two groups showed no statistically significant differences in any baseline characteristics, which confirmed that the covariate balancing was effectively achieved.

**Table 1:** Baseline characteristics before and after PSM

	Before propensity matching			After propensity matching		
Characteristics	C-PVP group	PVP group	*p*-value	C-PVP group	PVP group	*p*-value
Number (n)	65	132		54	54	
Sex (male/female)	5/60	25/107	0.04	5/49	10/44	0.13
Age (y)	68.72 ± 7.65	67.14 ± 8.08	0.18	67.54 ± 7.67	67.48 ± 7.92	0.97
BMI	23.81 ± 4.86	22.96 ± 3.41	0.21	23.29 ± 3.01	23.24 ± 2.81	0.91
BMD T-Value	-3.3 (-4.1~-2.8)	-2.8 (-3.7~-2.6)	< 0.01	-3.1 (-3.7~-2.8)	-3 (-3.9~-2.7)	0.36
Initial VAS scores	8 (7 ~ 8)	7 (7 ~ 8)	0.56	7.5 (7 ~ 8)	8 (7 ~ 8)	0.69
Initial ODI scores	75.72 ± 8.31	75.82 ± 6.89	0.94	76.33 ± 7.86	75.67 ± 6.89	0.64
Initial LKA (°)	11 (8 ~ 15)	11 (9 ~ 15)	0.85	11 (9 ~ 15)	11 (9 ~ 15)	1.00
Initial AVHR (%)	68.93 ± 11.15	68.78 ± 13.72	0.93	68.91 ± 10.79	67.39 ± 12.03	0.54
Initial MVHR (%)	62.31 ± 13.06	62.27 ± 10.80	0.99	62.78 ± 12.28	62.20 ± 10.37	0.81
Initial PVHR (%)	96.8 (93.3 ~ 98.4)	96.55 (93.42 ~ 98.25)	0.31	95.85 (92.3 ~ 97.47)	95.75 (92.6 ~ 97.18)	0.80
Fracture Type (n/%)			0.71			0.80
Mild (20–25%)	6 (9.23)	14 (10.61)		5 (9.26)	3 (5.56)	
Moderate (25–40%)	55 (84.62)	111 (84.09)		46 (85.19)	49 (90.74)	
Severe (> 40%)	4 (6.15)	7 (5.30)		3 (5.56)	2 (3.70)	
Fracture Segment (n/%)			0.65			0.68
Lumbar	8 (12.31)	23 (17.42)		7 (12.96)	6 (11.11)	
Thoracolumbar- Junction	53 (81.54)	101 (76.52)		43 (79.63)	46 (85.19)	
Thoracic	4 (6.15)	8 (6.06)		4 (7.41)	2 (3.70)	
Comorbidity						
CAD	2	5	0.80	2	2	1.00
HTN	26	44	0.36	20	20	1.00
COPD	1	3	0.73	1	2	1.00
DM	4	12	0.48	4	2	0.63
Neurologic disorders	6	11	0.83	5	6	1.00

Normally distributed continuous variables were summarized as mean ± standard deviation (SD). For continuous variables that did not follow a normal distribution, data were presented as median (interquartile range, IQR). Abbreviations: CAD, coronary artery disease; HTN, hypertension; COPD, chronic obstructive pulmonary disease; DM, diabetes mellitus

### Primary outcome

**Fig. 3:**
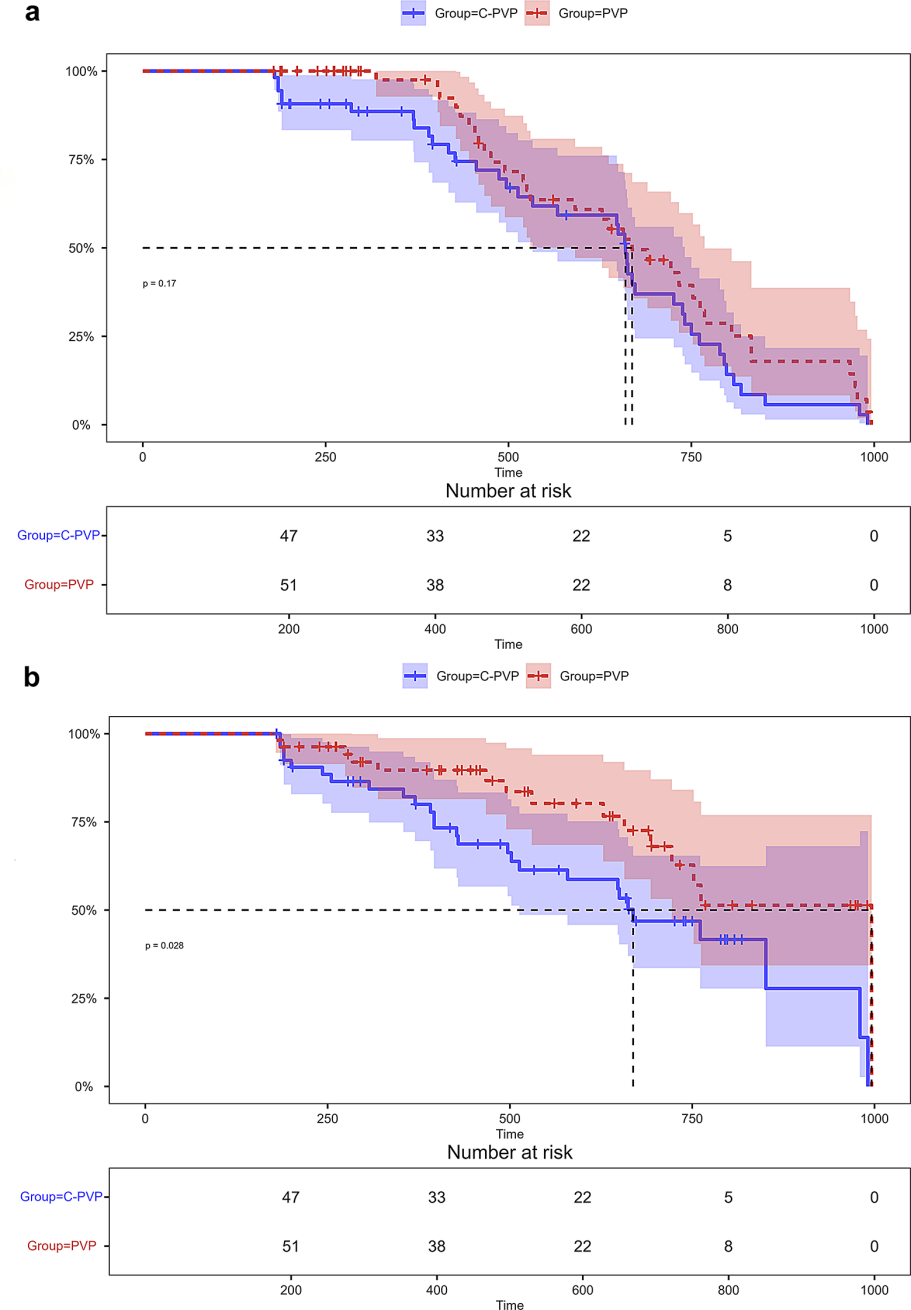
(**a**) Kaplan-Meier curves for VAS score incomplete pain relief time. (**b**) Kaplan-Meier curves for ODI score incomplete functional improvement time

The primary outcome measures were the VAS scores for pain relief and the ODI scores for functional recovery. As shown in Table 2, before the surgery, there were no statistically significant differences in VAS and ODI scores between the two groups. Both groups showed significant improvements in VAS and ODI scores 24 h after surgery. Although the C-PVP group exhibited greater reductions than the PVP group, these differences were not statistically significant. One month after surgery, the C-PVP group showed a significantly lower VAS score, 3 (3–3) compared to 3 (3–4) (*p* < 0.001), and ODI score, 28 (28–30) compared to 32 (30–34) (*p* < 0.001). The same trend of improvement was seen at six months postoperatively, with VAS and ODI scores being significantly better. These results confirm the better effectiveness of the C-PVP procedure in a medium- to long-term follow-up. At the final follow-up, the C-PVP group maintained significantly lower ODI scores (11.26 ± 4.38 vs. 14.11 ± 5.87, *p* = 0.008), although VAS scores were comparable between the groups (*p* = 0.24). In addition, a Kaplan–Meier analysis was conducted to address the effects of time confounding at the final follow-up. On the Kaplan-Meier plots, significantly higher numbers of patients treated with C-PVP obtained and maintained a VAS score of ≤ 1 or an ODI score of ≤ 10 at follow-up. The Kaplan-Meier curves indicated a higher probability of achieving complete pain relief over time in the C-PVP group compared to the PVP group. However, this difference was not statistically significant (*p* = 0.17) (Fig. 3a). Regarding functional outcomes, the C-PVP group showed a significantly higher probability of improvement over time (*p* = 0.028) (Fig. 3b). Number-at-risk tables showed a similar length of follow-up for both patient populations, indicating the long-lasting advantages of the procedures. These results underlined the greater efficacy of the C-PVP procedure in the long-term follow-up.

**Table 2:** Comparison of pain and functional efficacy between the two groups at each time point

Characteristics	C-PVP group	PVP group	t/Z statistic	*p*-value
VAS scores				
Pre-surgery	7.5 (7–8)	8 (7–8)	*Z* = 7.37	0.69
24 h post-surgery	4 (4–4)	4 (4–4)	*Z* = 8.16	0.33
1 month post-surgery	3 (3–3)	3 (3–4)	*Z* = 8.18	< 0.001
6 months post-surgery	2 (2–3)	3 (2–3)	*Z* = 8.07	0.02
Final follow-up	1 (1–2)	1 (1–2)	*Z* = 6.74	0.24
ODI scores				
Pre-surgery	76.33 ± 7.86	75.67 ± 6.89	*t* = 0.47	0.64
24 h post-surgery	36 (34–38)	37 (34–40)	*Z* = 6.50	0.12
1 month post-surgery	28 (28–30)	32 (30–34)	*Z* = 7.76	< 0.001
6 months post-surgery	18 (16–20)	20 (18–24)	*Z* = 6.29	0.02
Final follow-up	11.26 ± 4.38	14.11 ± 5.87	*t* = -2.76	0.008

Abbreviations VAS, Visual Analog Scale; ODI, Oswestry Disability Index

### Secondary outcome

#### Radiological parameters

Radiological parameters (LKA, AVHR, MVHR, and PVHR) were assessed pre-surgery and 24 h post-surgery. No significant differences in these parameters were observed between the two groups either before or after surgery (Table 3).

**Table 3:** Radiological parameters between the two groups

Characteristics	C-PVP group	PVP group	t/Z statistic	*p*-value
LKA (°)				
Pre-surgery	11.69 ± 3.81	11.74 ± 4.09	*t* = -0.07	0.95
24 h post-surgery	8 (7–11)	8.5 (7-11.75)	*Z* = 5.15	0.69
AVHR (%)				
Pre-surgery	68.91 ± 10.79	67.39 ± 12.03	*t* = 0.62	0.54
24 h post-surgery	75.17 ± 9.79	72.24 ± 11.82	*t* = 1.36	0.18
MVHR (%)				
Pre-surgery	62.78 ± 12.28	62.20 ± 10.37	*t* = 0.25	0.81
24 h post-surgery	73.11 ± 8.53	71.57 ± 10.23	*t* = 0.84	0.40
PVHR (%)				
Pre-surgery	94.09 ± 4.93	94.32 ± 4.75	*t* = -0.26	0.80
24 h post-surgery	97.25 (93.35–98.6)	96.3 (93-97.2)	*Z* = 3.23	0.10

Abbreviations: LKA, local kyphosis angle anterior; AVHR, anterior vertebral height ratio; MVHR, middle vertebral height ratio; PVHR, posterior vertebral height ratio

### Bone cement distribution

**Fig. 4:**
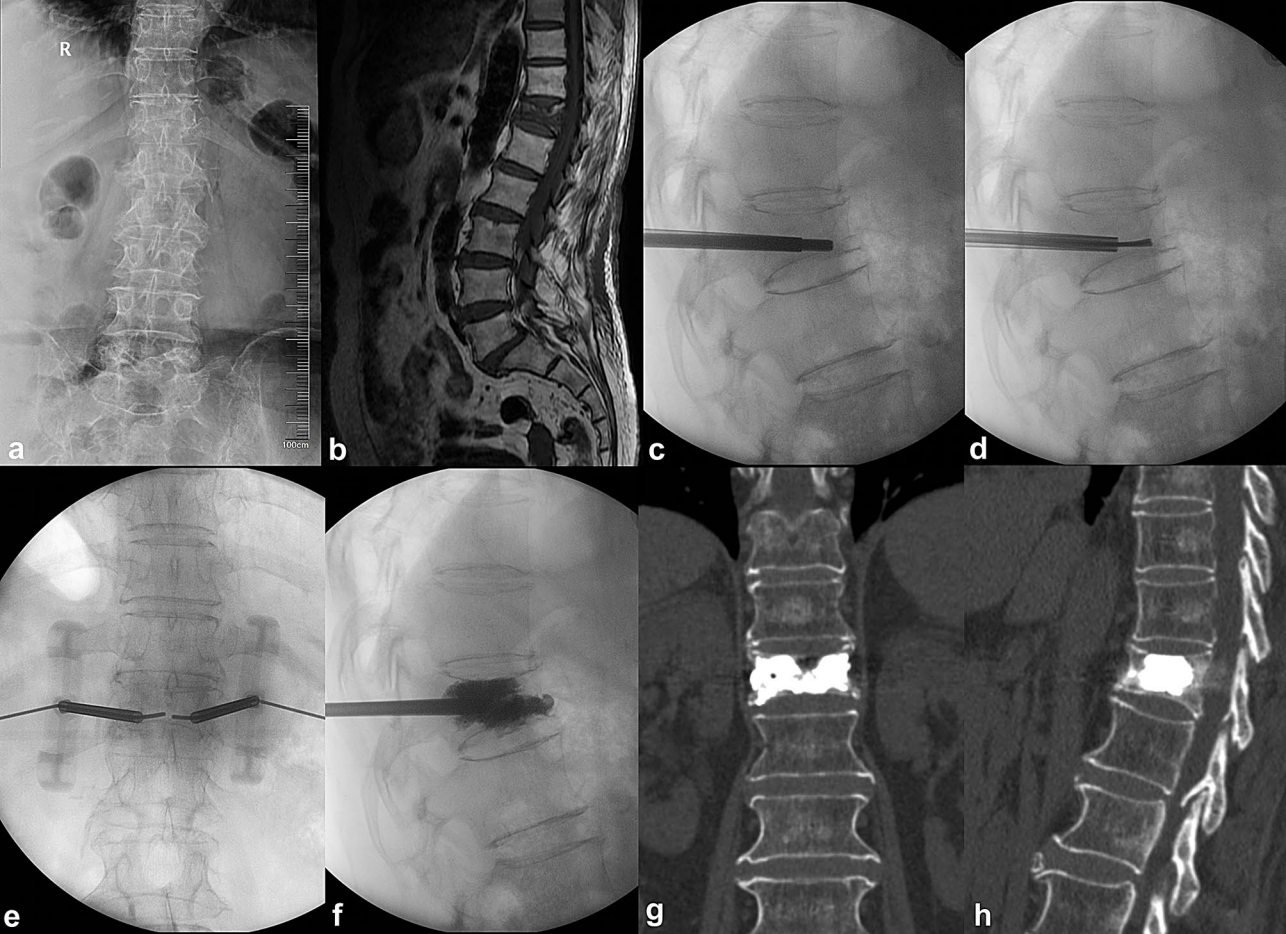
Typical case: An 82-year-old female patient with OVCF of T12 treated with C-PVP. (**a**) Preoperative X-ray. (**b**) Preoperative MRI. (**c-f**) Intraoperative fluoroscopic images illustrating the surgical procedure. (**g-h**) Postoperative three-dimensional CT

Bone cement distribution was assessed based on radiographic appearance and scored from 0 to 8 points. Better results were obtained in the C-PVP group, with higher scores of bone cement distribution. Up to 61.11% of patients in the C-PVP group achieved the optimal score of 8 in bone cement distribution, while only 24.07% in the PVP group did so (*p* < 0.001) (Table 4). A typical case is shown in Fig. 4.

**Table 4:** Bone cement distribution between the two groups

Groups	3	4	5	6	7	8	Z statistic	*p*-value
C-PVP group	1 (1.85)	2 (3.70)	1 (1.85)	6 (11.11)	11 (20.37)	33 (61.11)	4.09	< 0.001
PVP group	2 (3.70)	7 (12.96)	8 (14.81)	14 (25.93)	10 (18.52)	13 (24.07)		

### General clinical outcomes

The bone cement injection volume was significantly higher in the C-PVP group, with 4.99 ± 1.06 ml, compared to the PVP group, with 4.52 ± 1.02 ml (*p* = 0.03). Bone cement leakage was less frequent in the C-PVP group compared with the PVP group, and this difference was statistically significant (*p* = 0.02) (Table 5). Adjacent vertebral fractures were observed less frequently in the C-PVP group (5.56%) than in the PVP group (20.37%, *p* = 0.04). Other parameters, such as fluoroscopy frequency and operation time, were comparable between the groups (*p* > 0.05). During the follow-up period, no severe complications, such as pulmonary embolism, epidural hematoma, or nerve injury, were recorded.

**Table 5:** General clinical outcomes

Characteristics	C-PVP group	PVP group	t/Z/χ^2^ statistic	*p*-value
Final Follow-up Time (m)	16.93 ± 7.05	16.89 ± 7.78	*t* = 0.02	0.98
Operation Time (min)	43 (35-56.5)	43 (35-64.25)	*Z* = 5.60	0.38
Bone Cement Injection Volume (ml)	4.99 ± 1.06	4.52 ± 1.02	*t* = 2.23	0.03
Fluoroscopy Frequency	34.93 ± 10.13	33.85 ± 9.89	*t* = 0.77	0.44
Bone Cement Leakage (n/%)			*χ*^*2*^ = 5.82	0.02
B_Type	3 (5.56)	13 (24.07)		
S_Type	1 (1.85)	0 (0.00)		
None	50 (92.59)	41 (75.93)		
Adjacent Vertebral Fracture (n/%)			*χ*^*2*^ = 4.02	0.04
Yes	3 (5.56)	11 (20.37)		
No	51 (94.44)	43 (79.63)		

## Discussion

### Explanation of results

The results of our study demonstrate the significant advantages of using a curved guide wire in C-PVP compared to traditional PVP. The C-PVP group showed significant improvements in pain relief and functional recovery. These improved results are due to two main factors: better bone cement distribution and increased injection volume. Using the curved guide wire, our results revealed better bone cement distribution within the vertebral body. In this regard, better distribution is vital since it increases the structural integrity and stability of the vertebral body, which is essential for pain relief and preventing further fractures. The average bone cement injection volume in C-PVP was significantly higher than in traditional PVP, with a reduction in the rate of cement leakage. Additionally, the incidence of adjacent vertebral fractures (AVFs) was significantly lower in the C-PVP group, likely due to the improved distribution reducing mechanical stress on surrounding vertebrae. This indicates that the curved guide wire allows for a more effective and safe cement injection, alleviating the pain caused by vertebral microfractures. Moreover, the curved guide wire was modified without requiring additional procedural surgical instruments, thereby reducing the economic burden on patients.

### Bone cement distribution

Bone cement distribution is a contributing factor to the outcomes of PVP. Ideal bone cement distribution in the vertebral body can significantly increase the structural integrity and stability of the treated vertebrae [16]. The optimal distribution should achieve symmetrical dispersion on both sides of the vertebral body’s midline. Additionally, studies indicated that bone cement should interact with both end plates of a vertebral body, making it more stable with less possibility of recompression and secondary fractures [17]. Furthermore, even distribution of bone cement within the cancellous bone would lower the risk of localized stress fractures in the adjacent vertebrae [18]. This uniform distribution leads to better clinical efficacy compared to blocky or irregular cement patterns. Researchers have used numerous methods to improve bone cement distribution during PVP. For example, a curved injection cannula allows for more flexible cement injection via a unipedicular approach, resulting in enhanced distribution across the midline of the vertebral body [19]. Another method involves the “O” puncture approach, which employs a lateral entry point to achieve symmetrical cement distribution and improve clinical outcomes [20]. In our study, we utilized a curved guide wire technique to significantly enhance bone cement distribution during PVP. During our surgical procedure, multiple diffusion channels within the vertebra’s body were created, allowing a more uniform spread of cement. By ensuring low-pressure injection, our technique reduces the risk of cement leakage while maximizing the coverage of vertebral fractures.

### Bone cement injection volume

The mechanisms of pain relief provided by PVP remain unclear. Mechanical stabilization of the fractured vertebra is the principal mechanism by which bone cement alleviates pain in vertebroplasty [21]. The injection of PMMA cement into the vertebral body repairs microfractures, enhances vertebral stability, and effectively prevents micromovements at the fracture site [22]. This process regulates the stimulation of local nociceptive nerve endings, thereby diminishing pain related to vertebral microfractures. The bone cement injection volume in PVP is directly proportional to better pain relief [23]. Pain reduction correlates with injection volume, as an increased volume enhanced the stability of the fractured vertebra [24]. Optimizing cement injection improves patient outcomes by achieving maximum pain relief while minimizing associated risks [25]. Traditionally, bone cement is passively distributed based on pressure after injection. Increasing the volume raises the risk of leakage, leading to several complications, such as embolism, nerve compression, and leakage into adjacent structures [26]. However, our technique uses a curved guide wire to actively create multiple diffusion channels in the injured vertebra. This process reduces local pressure during cement injection, maintaining a low-pressure diffusion environment for the bone cement. Consequently, our study increased the injection volume without raising the leakage rate, which may be a reason for the enhanced clinical outcomes.

### Limitations

The present study is subject to several limitations that should be acknowledged. Firstly, the single-center design limits the generalizability of the findings, which may not be applicable across various demographic and clinical setups, potentially limiting the results’ external validity. Additionally, the retrospective nature of the study may introduce biases in participant selection and data collection. Although PSM was used to help balance baseline characteristics, residual confounding may still exist and impact the internal validity of this study. Moreover, the sample size was relatively small, particularly after matching, with 54 patients in each group, affecting affects statistical power. Furthermore, the use of telephone follow-up for tracking adjacent vertebral fractures may introduce recall bias, as it relies on subjective memory without confirmation through imaging, thus limiting the accuracy of patient-reported outcomes. Finally, the follow-up time varied among patients from 6 to 32 months. Even though Kaplan-Meier analysis was applied, potential temporal confounding effects may still be present.

### Conclusion

Overall, our study revealed that the use of a curved guide wire significantly enhanced bone cement distribution during PVP, thereby improving clinical efficacy in patients with OVCF. This technique provides an alternative to standard PVP treatment that is not only economical and safe but also effective for treating patients with OVCF.

## Data Availability

Data supporting the findings in this study are available from the authors, but some restrictions apply to the availability of the data. Considering the protection of patient privacy, the data are not publicly available. Upon reasonable request and with permission from the Clinical Ethics Committee of Anhui No. 2 Provincial People’s Hospital, the data can be obtained from the corresponding author.

## Abbreviations

OVCF: Osteoporotic vertebral compression fracture
PVP: Percutaneous vertebroplasty
C-PVP: Curved guide wire percutaneous vertebroplasty
VAS: Visual analog scale
ODI: Oswestry disability index
LKA: Local kyphosis angle
AVHR: Anterior vertebral height ratio
MVHR: Middle vertebral height ratio
PVHR: Posterior vertebral height ratio
BMD: Bone Mineral Density
PSM: Propensity Score Matching
HTN: Hypertension
CAD: coronary artery disease
COPD: Chronic Obstructive Pulmonary Disease
DM: Diabetes Mellitus
PMMA: Polymethylmethacrylate
